# Endoscopic surgery of a cavernous hemangioma in the external auditory canal

**DOI:** 10.1093/jscr/rjac242

**Published:** 2022-05-27

**Authors:** Myung Ho Jin, Min Young Kwak

**Affiliations:** Department of Otolaryngology-Head and Neck Surgery, Daejeon Eulji Medical Center, Eulji University School of Medicine, Daejeon, Republic of Korea; Department of Otolaryngology-Head and Neck Surgery, Daejeon Eulji Medical Center, Eulji University School of Medicine, Daejeon, Republic of Korea

## Abstract

Cavernous hemangioma of the external auditory canal (EAC) is a rare clinical finding. Endoscopic ear surgery has been successfully applied for various ear pathologies with a wider surgical sight, minimal canal cuts and precise manipulation of soft tissues. We report a case of a 60-year-old woman with hemangioma of EAC which increased in size without treatment for several years. She underwent excision of the hemangioma using transcanal endoscopic approach. The endoscopic transcanal approach allowed surgical resection of EAC cavernous hemangioma with wider views and minimally invasive techniques.

## INTRODUCTION

Hemangiomas are benign vascular tumors that occur most frequently during infancy and mainly affect the head and neck. There are several types of hemangiomas based on histopathological findings. These can be classified into capillary, venous and cavernous subtypes. Capillary hemangiomas are made up of numerous small capillaries that are normal in size and diameter. Venous or cavernous hemangiomas are composed of larger vascular spaces that are dilated and occur more frequently after the sixth decade of life.

Hemangiomas in the temporal bone rarely occur, and a hemangioma in the external auditory canal (EAC) is even more rarely reported in the literature. People with EAC hemangioma present with symptoms, such as hearing loss, pulsatile tinnitus, otorrhea, otalgia, ear fullness and facial palsy, depending on the location or size of the tumor. EAC hemangioma appears as an abnormal cluster of tightly packed vessels that are prone to bleeding. In a recent systemic review of EAC hemangiomas, most cases favored surgical excision with microscopic surgery using an endaural or postauricular approach.

In this paper, we report a case of EAC hemangioma excised by transcanal endoscopic ear surgery (TEES) after being only observed for several years.

## CASE REPORT

**Figure 1:**
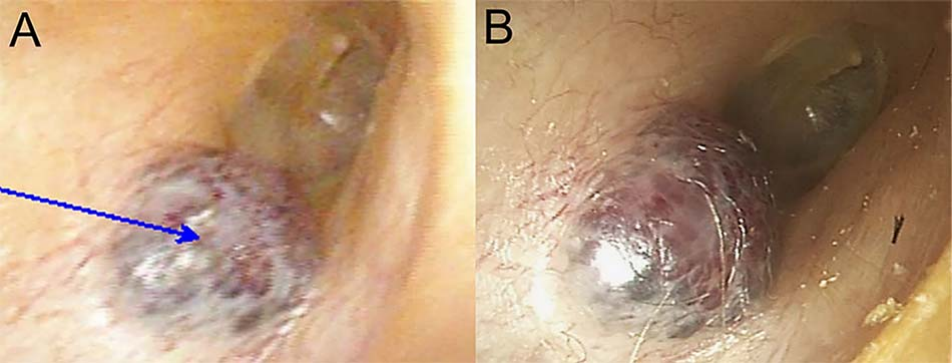
Otoendoscopic view of reddish, soft mass in the EAC; (**A**) at the time of the initial endoscopic examination; (**B**) 3 years after the initial endoscopic examination; a tumor had grown slowly, extending medially.

**Figure 2:**
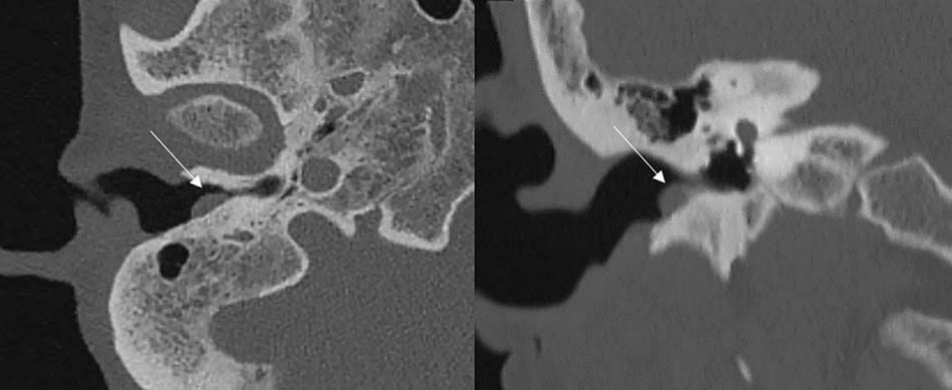
Axial and coronal CT scans showing a soft tissue mass (marked by the arrows) in the EAC without involvement of the tympanic membrane.

**Figure 3:**
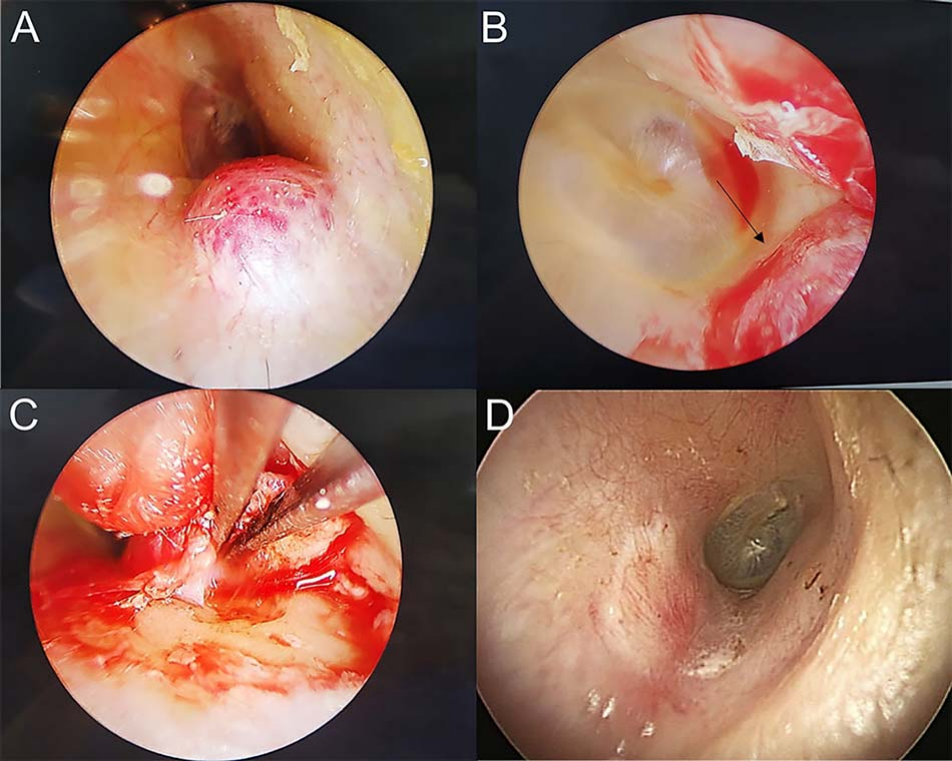
(**A**) Mass in right EAC; (**B**) Medial border (marked by arrow) of the mass near tympanic membrane was observed by endoscope; (**C**) excised mass and cauterized the feeding vessel with bipolar cautery; (**D**) 3 months after surgery, the EAC was covered by the perichondrium graft.

**Figure 4:**
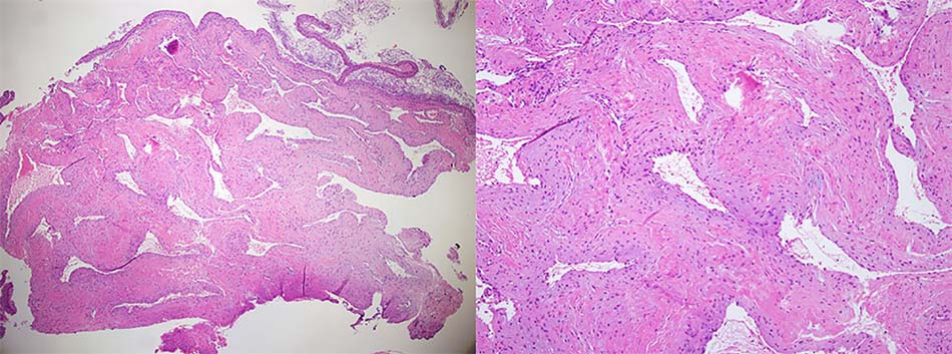
Histopathological image showing features of cavernous hemangioma (H&E stain, ×40, ×100).

A 60-year-old woman visited an otolaryngologic clinic due to itching and intermittent pain in the right ear that persisted for a month. Otoscopic examination showed a red tumor with a lobulated margin in the right EAC (Fig. 1A). She had a medical history of chronic renal failure, with no previous ear trauma or surgery. She was lost to follow-up for further evaluation after the first visit; however, she was re-visiting after 3 years. The patient did not report any symptoms, such as otalgia, fullness of the ears, dizziness, otorrhea, pulsatile tinnitus or hearing loss. The tumor had increased in size and had grown from a flat tumor into a round, bulging tumor close to the tympanic membrane (Fig. 1B). High-resolution non-contrast computed tomography (CT) imaging of the temporal bone revealed an 8.1 × 5.8-mm soft tissue mass in the posteroinferior wall of the right external ear (Fig. 2). The tumor was 3.1 to 4.0 mm away from the inferior annulus of the tympanic membrane. There was no evidence of bone erosion and the tympanic membrane and the middle ear appeared normal. The patient underwent endoscopic resection of the tumor via a transcanal endoscopic approach (Fig. 3). The inferior EAC mass was excised in bloc by elevating the skin in continuity with the lesion. The healthy surrounding skin 2 mm from the tumor margin was also removed. Bleeding was controlled by bipolar cautery. No significant bleeding was encountered. The skin defect in the ear canal was reconstructed by tragal perichondrium. Pathologic examination of the sample revealed the diagnostic features of cavernous hemangioma, with vascular spaces lined by a single layer of flat endothelial cells without atypia or mitosis (Fig. 4). The patient healed well after surgery, with no sign of recurrence after 10 months.

## DISCUSSION

Hemangiomas are benign vascular tumors that most commonly affect the head and neck region and are highly correlated with embryologic fusion lines. Hemangiomas involving the EAC are rare. In a recent systemic review in 2021, 38 cases of hemangioma of EAC were reported between 1972 and 2020, and cavernous hemangiomas represented 57% of the reported cases [1].

Preoperative imaging may help confirm the diagnosis of EAC hemangioma and rule out other otologic disorders. In previous reports, temporal CT combined with magnetic resonance imaging (MRI) was commonly used in evaluating vascular tumors in the EAC. Temporal CT is the first choice to evaluate these lesions, and EAC hemangiomas appear as isodense lesions. In contrast-enhanced CT, these lesions have no or poor enhancement [2, 3]. MRI is occasionally performed for some vascular lesions involving the EAC, but it is not necessary unless the tympanic cavity or mastoid is involved. Preoperative embolization was done in advanced cases; however, it is not essential for all [4]. In the present case, contrast enhancement was avoided due to her chronic kidney disease, and non-contrast CT alone was sufficient to identify an isolated EAC hematoma.

In most cases, these lesions were described as smooth multilobulated tumors with a color varying from bright red to violaceous [5]. Suspected asymptomatic EAC hemangiomas may not require surgical treatment but may need to be monitored for any changes in size. The careful observation of tumor lesions in the EAC is necessary because clinicians may misinterpret malignant conditions that require histopathological confirmation. Most advocate surgical resection of EAC hemangiomas because an unidentified or untreated small hemangioma that occupies a small and enclosed ear canal becomes problematic even though it grows slightly.

Hemangiomas in other regions of the head and neck regions either proliferate or regress [6]. A spontaneous disappearance usually occurs in capillary hemangiomas during childhood, but cavernous hemangiomas are less likely to regress [7]. Few studies have worked to provide details related to the natural progression of EAC hemangiomas. In a case reported by Balkany *et al*., tumor size doubled in 18 months, whereas tumors did not grow in 6–15 months of follow-up in two cases reported by Magliulo *et al*. [8]. De Zinis *et al*. observed two adults with benign vascular lesions for 4 and 10 years, respectively, and their sizes did not change [5]. In our case, the tumor size grew slightly from a flat tumor to a bulging tumor in 3 years.

Surgical approaches are considered depending on the size and location of the tumor as well as the degree of hearing loss. Small lesions can be removed using endaural and transcanal approaches, whereas in advanced lesions, the transmastoid or middle fossa approach or combined methods must be considered. To date, endaural or postauricular approaches using microscopic approaches for most hemangiomas with EAC have been described in the literature [1]. In our case, minimally invasive TEES was preferred. Compared to the microscopic approach, endoscopic resection can help observe the medial margin of EAC masses near the tympanic membrane, which often becomes a blind spot of the microscope with a minimally invasive approach. Today, TEES has been performed more and more due to its benefits, including wider views, improved imaging quality and a minimally invasive procedure [9]. TEES with a clear surgical view and precise manipulation is beneficial for the complete resection of EAC hemangiomas. Except for advanced cases, EAC hemangiomas are usually small (5.0–15.0 mm) and less bleeding is observed [2, 10]. TEES can also be a treatment option to identify the precise margin of EAC hemangiomas. Although TEES was performed during general anesthesia in our case, local anesthesia appears to provide more benefits and convenience for patients.

The recurrence rate after surgical resection is not fully understood because acquired EAC hemangioma is a very rare entity. According to a recent systematic review of EAC hemangiomas, most cases (31/38, 81.5%) had no recurrence [1]. Four cases of recurrence of ear hemangiomas from the other previous reports were related to the adequacy of surgical resection [11–14]. Recurrence after surgical treatment of hemangiomas in other parts of the body is also related to their complete resection [15]. Therefore, complete resection with precise observation using endoscopy can be recommended in selective cases of EAC hemangioma.

## CONCLUSIONS

We present a case of an EAC hemangioma resected using an endoscopic approach, which provides improved wide views without blind spots. There were no intraoperative bleeding or other complications. This approach is minimally invasive and provides improved visualization compared to standard microscopic resection.

## DATA AVAILABILITY

The data are not available for public access because of patient privacy concerns.

## AUTHORS’ CONTRIBUTIONS

M.Y.K. and M.H.J. performed the operation and management of the patient in this case report. All authors read and approved the final manuscript.
